# Multistate Outbreak of MDR TB Identified by Genotype Cluster Investigation

**DOI:** 10.3201/eid1801.110671

**Published:** 2012-01

**Authors:** Pennan M. Barry, Tracie J. Gardner, Elizabeth Funk, Eyal Oren, Kimberly Field, Tambi Shaw, Adam J. Langer

**Affiliations:** California Department of Public Health, Richmond, California, USA (P.M. Barry, T. Shaw);; Alaska Division of Public Health, Anchorage, Alaska, USA (T.J. Gardner, E. Funk);; Centers for Disease Control and Prevention, Atlanta, Georgia, USA (T.J. Gardner, A.J. Langer);; Public Health—Seattle and King County, Seattle, Washington, USA (E. Oren);; Washington State Department of Health, Olympia, Washington, USA (K. Field)

**Keywords:** MDR TB, tuberculosis, multidrug resistant, cluster investigation, genotype, genotype cluster, Alaska, California, Washington, bacteria, tuberculosis and other mycobacteria, antimicrobial resistance

## Abstract

In 2008, diagnosis and investigation of 2 multidrug-resistant tuberculosis cases with matching genotypes led to identification of an outbreak among foreign-born persons who performed short-term seafood production work in Alaska during 2006. Tuberculosis control programs should consider the possibility of domestic transmission even among foreign-born patients.

In the United States, 60% of tuberculosis (TB) cases occur among foreign-born persons (*1*). Infection is often assumed to be acquired before immigration. However, many foreign-born persons have risk factors for acquiring TB domestically, such as living and working in crowded conditions with persons at higher risk for having TB (*2*). With the nationwide implementation of universal TB genotyping through the National TB Genotyping Service (NTGS) (*3*), previously unknown outbreaks can be identified. We describe an outbreak of multidrug-resistant (MDR) TB among foreign-born migrant workers that was identified by genotype cluster investigation.

## The Study

During 2009, the California Department of Public Health became aware of MDR TB cases with a matching genotype and drug-resistance pattern (resistant to isoniazid, rifampin, ethionamide, and streptomycin) in 2 foreign-born patients (designated CA1 and CA4). The cases were diagnosed during 2008 in adjoining California counties; 1 patient was born in Asia, the other in Latin America. Review of the patients’ activities and lists of contacts did not expose commonalities. For 2004–2009, the NTGS database contained 1 other case in the United States with a matching genotype. This case, in an Africa-born patient (WA1), was diagnosed in Washington, USA, in 2008. Sputum smear results were positive, and the drug-resistance pattern matched that of the other 2 cases-patients. Further investigation, including repeat interviews, showed that all 3 case-patients had a history of short-term seafood production work in Alaska.

The California Department of Public Health notified the Alaska Division of Public Health (ADPH) about this suspected MDR TB transmission in Alaska. ADPH reviewed case records and identified an Africa-born patient (AK1) with MDR TB and positive sputum smear results who had been employed in seafood production at the time of his 2006 diagnosis. During contact investigation for the case, ADPH evaluated 3 roommates with previously positive tuberculin skin test results. ADPH did not expand the contact investigation because many workplace contacts were no longer employed at the facility and were unreachable. No persons identified in the initial contact investigation were subsequently identified as outbreak case-patients. A 2010 review of employer records confirmed that all 4 case-patients had been employed in the same facility during AK1’s infectious period (Figure). Because contact information for other workers was unknown, no further investigation could be pursued.

**Figure:**
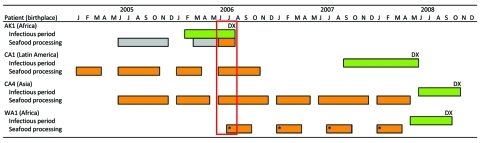
Infectious periods and work schedules for 4 multidrug-resistant tuberculosis–infected seafood production workers (AK1, CA1, CA4, and WA1) with matching mycobacterial genotypes, Alaska, USA, 2005–2008. Green, infectious period; orange, work at processing facility A; gray, work at another processing facility; red box, likely period of transmission. DX, date of diagnosis. *Approximate time periods.

Initial genotyping results (spoligotyping and 12-locus mycobacterial interspersed repetitive units–variable number of tandem repeats [MIRU-VNTR] analysis) demonstrated that AK1’s isolate had a genotype that differed from those in California and Washington at 1 MIRU-VNTR locus (*4*). To confirm these results and further evaluate the relatedness of the isolates, 24-locus MIRU-VNTR (*5*) and IS*6110*-based restriction fragment length polymorphism analyses (*6*) were conducted and showed exact matches among the 4 cases by spoligotype, 24-locus MIRU-VNTR, and restriction fragment length polymorphism (Table 1). The single-locus difference in initial and subsequent genotype results of AK1 was determined to be a laboratory error.

**Table 1:** Genotyping of MDR TB spoligotype 477777777720771 among case-patients linked to seafood production work, Alaska, USA, 2005–2008*

Locus	No. repeats
Miru02	2
Miru04	2
Miru10	7
Miru16	3
Miru20	2
Miru23	5
Miru24	1
Miru26	5
Miru27	3
Miru31	3
Miru39	2
Miru40	3
424	2
577	3
1955	3
2163b	5
2165	3
2347	4
2401	4
2461	2
3171	3
3690	3
4156	3
4052	5

*Determined by using 24-locus mycobacterial interspersed repetitive units–variable number of tandem repeats. MDR TB, multidrug-resistant tuberculosis.

The 4 case-patients worked in a seafood production facility in Alaska during the summer of 2006. The facility included multiple large buildings with high ceilings and open areas with production lines. Patients AK1, CA1, and CA4 completed follow-up interviews about their activities in Alaska. All 3 reported working in the same building; 2 also worked in a second building. They lived in 3 different apartments that were in 2 different buildings and ate and socialized primarily in each dormitory’s cafeteria. Case-patients reported working up to 12 hours per day, 7 days per week; they did not report any common activities outside of work. No other links were identified among the case-patients.

Contact investigation of the secondary cases (CA1, CA4, and WA1) was conducted, and 47 (96%) of 49 contacts were fully evaluated. Of these 47 contacts, 2 had active TB disease (CA2 and CA3, US-born children with negative culture results), and 30 (64%) had latent TB infection (LTBI); 28 began treatment for MDR LTBI (Table 2). Review of these contact investigations determined that reopening and expanding the investigations would not be productive.

**Table 2:** Results of initial contact investigations of MDR TB cases linked to seafood production work, Alaska, USA, 2006–2008*

Contacts	No. contacts, by case-patient				Total no. contacts
	AK1†	CA1‡	CA4§	WA1¶	
Total no. contacts identified	3	33	10	3#	49
Total no. contacts evaluated	3	33	10	1	47
Active TB	0	2**	0	0	2
LTBI	3	18	8	1	30
TST positive ††	3	9	8	1	21
TST conversion‡‡	0	9	0	0	9
Started on LTBI treatment§§	0	17	10	1	28
TST negative	0	13	2	0	15

*MDR TB, multidrug-resistant tuberculosis; ID, identification; AFB, acid-fast bacilli; LTBI, latent TB infection; TST, tuberculin skin test. †Source patient. Patient had positive sputum AFB smear results, and initial chest radiograph did not show cavitary disease. ‡Patient had positive sputum AFB smear results, and initial chest radiograph showed cavitary disease. §Patient had negative sputum AFB smear results, and initial chest radiograph did not show cavitary disease. ¶Patient had positive sputum AFB smear results, and initial chest radiograph showed cavitary disease. #An active TB case was later identified; the patient had a matching genotype and an epidemiologic link to WA1, but this tertiary case was not identified during the secondary case-patient’s contact investigation. **Contacts are children (CA2 and CA3) with negative culture results. ††Includes persons for whom time of conversion was not known (i.e., prior positive or new positive TST). ‡‡Defined as a positive TST following a negative TST performed in preceding 2 y. §§Includes 2 children <5 y of age with initial negative TSTs started on “window” LTBI treatment while awaiting follow-up TST.

To facilitate prompt investigation, the Centers for Disease Control and Prevention is now actively monitoring the NTGS database for new cases matching the outbreak genotype. NTGS surveillance identified 1 additional case in Washington State during 2010. This case-patient had never worked in seafood production and had not been identified during the contact investigation of WA1. However, his cousin was WA1’s roommate, and he later moved into WA1’s apartment after WA1 moved out, so unrecognized contact could have occurred.

Genotype cluster investigation showed previously unrecognized domestic transmission of MDR TB among foreign-born migrant workers. Investigation identified 7 MDR TB cases: the probable source case in Alaska, 3 secondary cases among co-workers in whom MDR TB subsequently was diagnosed elsewhere, and 3 tertiary cases among contacts of the secondary cases. Transmission probably occurred while the case-patients were working.

## Conclusions

The initial contact investigation of the presumed source case was limited by the remote location and the short-term nature of the employment. In addition, a high rate of previously positive tuberculin skin test results among close contacts made the degree of transmission among that group impossible to assess. In similar circumstances, expanding contact investigations of patients with positive sputum smear results beyond the initial group of contacts might be productive. The use of interferon-γ release assays to test samples from contacts who have received *Mycobacterium bovis* BCG might help assess the degree of transmission (*7*).

This outbreak underscores the importance of considering TB transmission in nonresidential settings. Current TB-control guidelines emphasize the need to identify and assess the risk for transmission at all possible sites, including workplaces (*8*). Local health departments should weigh the probable yield of expanded and worksite investigations relative to other TB-control activities. If worksite investigations are pursued, clarifying employers’ responsibilities for funding and supporting those investigations might help mobilization of the substantial resources typically required.

Industries that employ large numbers of foreign-born workers from countries with a high TB incidence might encounter TB among their employees. In seafood production facilities, where those workers live and work together, transmission risk is likely increased. Interventions to identify TB cases more quickly include employee and employer education regarding TB symptoms and institution of a cough alert program to ensure access to clinical evaluation of a persistent cough (*9**,**10*). TB control programs should consider the possibility of domestic TB transmission even among foreign-born patients, particularly if the patients have lived or worked in crowded conditions with other persons at higher risk of having TB.

Because initial genotype results indicated an exact match among only 3 of the 4 cases, an additional conclusion of this outbreak is that epidemiologic links are possible among case-patients with closely related genotypes. However, resources to explore epidemiologic links among patients with nonmatching genotypes should be used judiciously. Discussion with the genotyping laboratory and retesting are important first steps when epidemiologic links are suspected among patients with closely related genotypes.

Although pre-employment TB screening and LTBI treatment is a strategy for preventing progression to TB among foreign-born persons, and the standard LTBI drug regimens used probably would have prevented an outbreak of drug-susceptible TB, that strategy would not have averted this MDR TB outbreak. A 1992 Centers for Disease Control and Prevention guideline on preventing and controlling TB among migrant farm workers prioritized screening asymptomatic workers for TB as an activity lower than diagnosing, treating, and performing contact investigation for cases of active TB (*2*). The costs and benefits of screening in this analogous population should be investigated.
